# Sliding down the socioeconomic health gradient of COVID-19 in New York City: multinomial regression analyses of disproportionate financial hardship for Black, Latino, and Asian residents and households with children

**DOI:** 10.3389/fpubh.2025.1603629

**Published:** 2025-09-02

**Authors:** Rose Jimenez, Sheena Dorvil, Jennifer Pierre, Christina Nieves, Lauren J. Shiman, Tanzia Shaheen, Rachel Dannefer, Shale Maulana, Nika Norvila

**Affiliations:** ^1^Bureau of Brooklyn Neighborhood Health, Center for Health Equity and Community Wellness, New York City Department of Health and Mental Hygiene, New York, NY, United States; ^2^Bureau of Harlem Neighborhood Health, Center for Health Equity and Community Wellness, New York City Department of Health and Mental Hygiene, New York, NY, United States; ^3^Bureau of Bronx Neighborhood Health, Center for Health Equity and Community Wellness, New York City Department of Health and Mental Hygiene, New York, NY, United States; ^4^Bureau of Chronic Disease Prevention, Center for Health Equity and Community Wellness, New York City Department of Health and Mental Hygiene, New York, NY, United States; ^5^NYC Health Panel, Center for Health Equity and Community Wellness, New York City Department of Health and Mental Hygiene, New York, NY, United States

**Keywords:** New York City, economic deprivation, socioeconomic health gradients, households with children, precarious employment, COVID-19, health equity, health geography

## Abstract

**Background:**

Distinct socioeconomic gradients in COVID-19 outcomes were observed across the United States, so an evaluation of individual resident characteristics related to economic deprivation (race or ethnicity, precarious employment, children in the household) was conducted to inform neighborhood reach strategies by the NYC Department of Health and Mental Hygiene.

**Methods:**

A cross-sectional survey was fielded to participants from a probability-based sample of South Bronx, North and Central Brooklyn, and East and Central Harlem residents. Responses rates for financial difficulty experienced since the pandemic onset were organized into three categories: “never” experiencing financial difficulty, or experiencing “short-term” or “prolonged” financial difficulty. Controlling for age, gender, birthplace, educational attainment, income level, employment, and financial assistance received, two multinomial logistic regression analyses were used to examine the prevalent association between race-ethnicity or household composition and the type of financial difficulty experienced.

**Results:**

We found that Black residents, Latino residents, residents with children in their household, and people living ≥200% below the poverty threshold were most likely to experience financial difficulty. Compared to non-Latino White residents, all other racial and ethnic groups were twice as likely to experience prolonged financial difficulty. Households with children were 40% less likely to avoid financial difficulty and 52% more likely to experience prolonged financial difficulty compared to those without.

**Conclusions:**

Delays and premature discontinuation of benefits were correlated to avoidable hardship to those in need. Government policy fosters the inequitable distribution of resources in the U.S. those policies continue to predispose vulnerable groups to harm through economic deprivation and racial residential segregation.

## 1 Introduction

**Figure 1:**
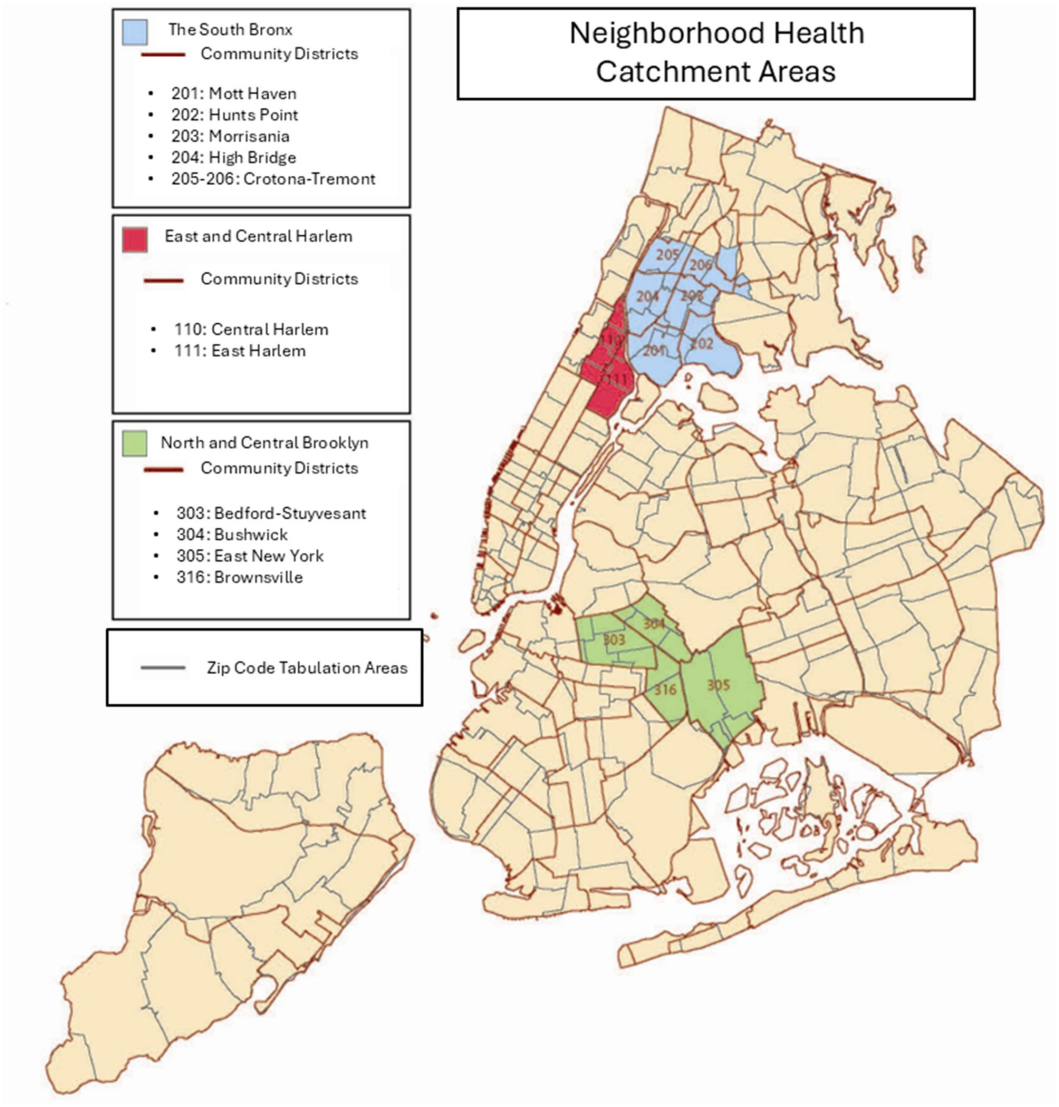
Map of South Bronx, Harlem, and Brooklyn Neighborhood Health Catchment areas in New York City, including zip code tabulation area boundaries and community district boundaries.

During the SARS-CoV-2 Coronavirus Disease pandemic of 2019 (COVID-19) (1), in addition to a profound loss of life, more than half of New York City (NYC) residents were affected by a loss of employment income in their household (2). Within this densely-populated city, there is a high level of racial residential segregation, and a history of environmental health hazards that disproportionately harm Black and Latino communities (3). The NYC Department of Health and Mental Hygiene (Health Department) operates three Bureaus of Neighborhood Health (BNH) to serve areas that bear undue health burdens, such as higher rates of chronic disease, respiratory disorders, and economic precarity as artifacts of structural racism: Catchment Areas in the South Bronx, North and Central Brooklyn, and East and Central Harlem in Manhattan (NYC Community District numbers 110, 111, 201 through 206, 303, 304, 305, and 316) (4). See Figure 1. These Catchment Areas were identified by the Health Department as being high-need because of the confluence of high health disparities (5) and the historical segregation and disinvestment city planning practices that primarily targeted Black and Latin Americans, such as redlining and racially restricted covenants, which effectively confined Black and Latin Americans to high-poverty enclaves. This study assimilates to these Catchment Areas to inform practices and policies for the City's neighborhood reach strategies in these neighborhoods.

At the onset of the pandemic, 8.24% of the labor force residing in these districts were unemployed (6), compared with a 2.80% unemployment citywide rate. Among all BNH residents, 29% were working in recreation, entertainment, retail, food service, hospitality, or other close-personal services: repairpersons, mechanics, hairdressers, other cosmetology services, laundry, work in private households, and religious services (7). The precarity of these industries was made evident when many of those workplaces were either shut down, subject to workforce reduction restrictions as outlined in New York State Executive Order 202.6, or otherwise stringently limited as precautions against viral transmission in March 2020 (8). Precarious employment such as this is a distinct health stressor because employment stability is a social determinant of health (9, 10).

Emerging research shows that there were socioeconomic gradients in COVID-19 outbreaks and clinical outcomes related to household composition in urban settings in the United States (U.S.) (11–13). In one study, households with children living in crowded or multi-unit housing had higher rates of SARS-CoV-2 acquisition and higher rates of COVID-like illness in early 2020 compared to both households without children and households with children in single-unit dwellings (14).Concurrent research also demonstrates increases in economic hardship in families with young children during the pandemic (15, 16).

In response to this nationwide economic crisis, a stimulus bill for Coronavirus Aid, Relief, and Economic Security (CARES) was enacted in March 2020. Under U.S. Public Law Number 116–136, the suite of direct eligibility-based individual economic relief included income-based Economic Impact Payments (EIP, or “stimulus checks”), and three programs that temporarily increased unemployment benefits. Additional relief measures included secured federal student aid, suspension of federal student loan repayment and interest accrual, waivers on early distribution and withdrawal penalties for retirement funds, moratoria on property foreclosure and eviction, and expansion of coverage options through the U.S. government national health insurance program Medicare (17).

In January 2021—10 months after workforce reduction restrictions were put in place by New York State—a federal order on economic relief from the pandemic was enacted (18). This American Rescue Plan mounted a national vaccination program and the following supplementary individual economic relief: additional EIP, extended unemployment benefit eligibility and insurance, emergency aid for rent back-payments, lowered health insurance premiums, and eligibility-based increased payouts for the Supplemental Nutrition Assistance Program, Earned Income Credits (EIC), Temporary Assistance to Needy Families, and Child Tax Credits (CTC) (19).

Despite these policies and injection of aid, a preceding assessment of healthcare utilization in BNH catchment areas from the same survey we evaluate in this paper found that residents experienced significant disruption to healthcare during the COVID-19 pandemic, where 14% of residents delayed routine physical healthcare due to cost, and 20% experienced sustained delayed mental healthcare due to lack of insurance (20). In the U.S., employment is not just a source of wages, it is also intimately tied to health insurance options, so precarious employment begets precarious healthcare access. Consistently, people with low household income are overrepresented among those reporting unmet needs that correlate to deteriorated future health. This suggests that unmet needs reflect “reduced access to needed health care, and therefore may have a role in assessing health system equity as a complement to utilization-based approaches,” (21) such as that in a previous assessment. Another study, using multinomial regression, showed that excess unmet needs, including unmet needs for healthcare, in 2020 as compared to prior time periods were attributable to COVID-19 conditions (22).

BNH areas and other NYC neighborhoods where service workers with high levels of direct contact interactions like home health aides, nurse aids, food service workers, and warehouse staff reside had high concentrations of positive COVID-19 cases (23). In this way, the various workplace transmission reduction guidelines effectively perpetuated community-based inequality by protecting more affluent areas where more workers who were able to work remotely and isolate or quarantine while others, like the food delivery workers serving work-from-home groups, were at higher risk of exposure.

The variation in COVID-19 mortality and morbidity across place showcase the persistence of health inequities and economic deprivation created through the mechanism of residential segregation (24, 25) and begs the question as to the extent of financial struggles occurring in BNH neighborhoods during this time.

To improve plans and policies for hyperlocal response during the remainder of COVID-19 and in future public health emergencies (26), the purpose of this study is to determine which BNH residents are the most vulnerable in events where rapid emergency funds disbursal is needed.

BNH neighborhoods are historically populated by Black and Latino residents and have low median household incomes. BNH reach strategies function through place-based interventions (4), but the racial, ethnic, age, and income-level composition of these neighborhoods are transforming through the processes of gentrification (27–29). Considering the increasing diversity of these areas, it is necessary to evaluate them on a more granular level (30).

In order for the NYC Health Department to continue to confront community health inequities in the South Bronx, North and Central Brooklyn, and East and Central Harlem that are rooted in historical neighborhood disinvestment, this work explores the extent to which financial stress varied among residents within these areas.

Demographic characteristics that are known to be related to social vulnerability include race or ethnicity, household composition, age, gender, U.S. nativity, individual educational attainment, household income level in relation to poverty, employment status, and precarious employment (31, 32). We hypothesized that these characteristics may be associated with experiencing financial difficulty from the onset of the pandemic in Spring 2020 to the time of the study in Fall 2021.

Specifically, we asked: During this period in BNH catchment areas, which socioeconomic characteristics are associated with experiencing any financial difficulty since the onset of the pandemic within BNH neighborhoods? What is the relative risk between race or ethnicity and experiencing short-term or prolonged financial difficulty among BNH residents during COVID-19? What is the relative risk between presence of children in the household and experiencing short-term or prolonged financial difficulty among BNH residents during COVID-19?

## 2 Materials and methods

### 2.1 Data

From September 30 to November 4, 2021, the BNHs conducted the COVID-19 Community Recovery study: a cross-sectional survey of NYC residents through the NYC Health Panel (known then as “Healthy NYC”). The survey could be self-administered online or conducted via Computer-Assisted Telephone Interviewing (CATI). It was available in English, Spanish and Chinese (Simplified Chinese online and Mandarin and Cantonese through CATI). It posed questions across six main domains: impact of the COVID-19 pandemic on general healthcare, prescriptions, and mental health; attitudes toward COVID-19 vaccines and knowledge of NYC COVID-19 testing services; perceived community resilience and assets needed for recovery; trust in local government; social determinants of health; and linkage to respondent's local BNH. This analysis focuses on response data captured from the social determinants of health domain—specifically, experiences with financial difficulty during the pandemic. We also include response data from the mental domain regarding the death of family members during this time, which may have had direct impact on family finances. This probability-based survey panel provided a timely approach to collecting population-based data on experiences among NYC residents. Sampling and recruitment for the panel has been detailed previously (33, 34).

Eligibility criteria for the COVID-19 Community Recovery survey included NYC Health Panel members aged 18 years or older living in one of the 12 districts or 25 ZIP code tabulation areas within BNH catchment areas. Out of 9,315 panelists, 4,478 panel members were deemed eligible for the study and invited by mail, email, or text message to participate in the survey. With a response rate of 30.3%, a total of 1,358 BNH residents, living in Harlem (*N* = 523), the Bronx (*N* = 434), and Brooklyn *(N* = 401) catchment areas participated in the COVID-19 Community Recovery Survey. To ensure data would inform trend analyses despite this differential nonresponse, for example, a high relative ratio of women to men, completed surveys were weighted to housing characteristics and calibrated to U.S. Census estimates by race, birth sex by age and borough, and education, resultantly making the data representative of adults residing in the BNH catchment areas (34).

### 2.2 Dependent variable

The depenent variable for this study was the experience of financial difficulty since the onset of the COVID-19 pandemic. This was determined by asking respondents whether they had experienced the following financial difficulties due to the COVID-19 pandemic: “Been unable to pay the rent or mortgage? Been unable to pay the gas, oil, or electricity bills? Been unable to pay the telephone (including cellphone) or internet bills? Been unable to afford subway or bus fare?” Those who answered “yes” to any of these survey items were considered to have experienced at least one financial difficulty since the pandemic began in March 2020. Those that experienced at least one financial difficulty were then asked whether they were still experiencing any of these problems as of the time of the survey in Fall 2021. Questions from this survey domain were adapted from the NYC Health Department Health Opinion Polls (HOP) and Community Health Survey (CHS), which are regularly fielded citywide.

**Figure 2:**
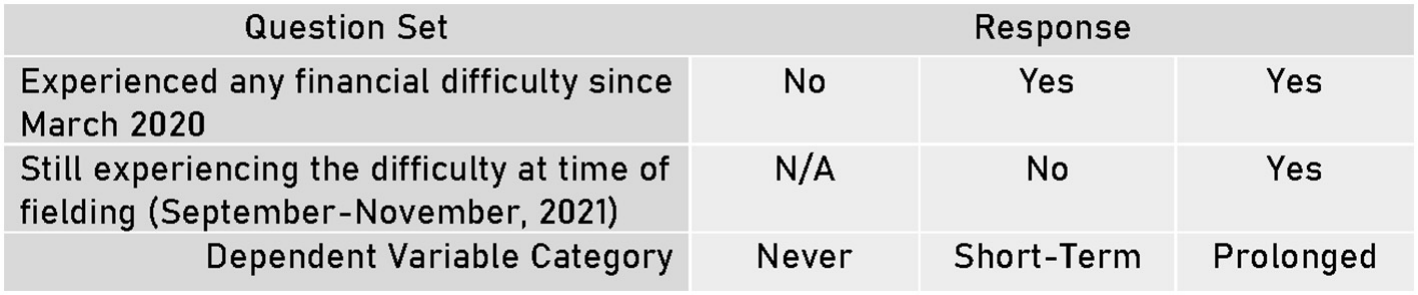
Determination of dependent variable category by participant response types.

For the multinomial regression analysis, the dependent variable was developed from the two aforementioned survey questions and separated into the following three nominal categories: “Never,” where the respondent reported never experiencing any of the listed financial difficulties from March 2020 through the time of taking the survey, “short-term” financial difficulty, where the respondent reported experiencing at least one financial difficulty since March 2020, but not experiencing any difficulties by the time of the survey in Fall 2021, and “prolonged” financial difficulty, where the respondent reported experiencing at least one financial difficulty since March 2020 and still experiencing the difficulty as of taking the survey in Fall 2021. As this is a multinomial regression analysis, each of these categories is treated as independent from one other. See Figure 2.

### 2.3 Independent variables

For this study we had two main independent variables: (1) race or ethnicity and (2) household composition. Race or ethnicity was determined through the combination of self-reported race and Latino ethnicity into the following categories: White non-Latino, Black non-Latino, Latino or Hispanic, Asian non-Latino, and Other or Multi-racial non-Latino. We acknowledge that race and ethnicity are both social constructs, which can be influenced by perceived physical characteristics, ancestry, and cultural identity (35). In this study, the race or ethnicity covariate serves as a proxy representation for the impacts of discrimination and structural racism. The household composition factor was defined through presence of children in the household where respondents reported whether one or more children aged 17 years or younger were living in their household.

The additional covariates were aggregated as follows. Self-disclosed gender was separated into three categories: (1) man, (2) woman, and (3) transgender man, transgender woman, non-binary person, or a gender not mentioned. Nativity was defined by place of birth relative to the U.S.: within the U.S., within U.S. territories, or outside the U.S. Individual educational attainment was categorized into less than a high school degree; high school graduate; some college, technical school, or associate degree; college graduate (four or more years); and graduate degree or professional degree. The household income level variable uses the federal poverty level (FPL) thresholds of below the 200% FPL and 200% or greater than the FPL. Employment status was divided into employed, unemployed, not in the labor force (defined as being a student, homemaker, retiree, or being unable to work), and more than one category. Respondents who selected more than one item were categorized into this last group.

Since employment status was collected during the NYC Health Panel registration process, we also measured whether respondents lost work, had reduced work hours, or had a pay decrease during the time frame of enrollment to the date of participation in the survey (March 2020 to October 2021) as a proxy for precarious employment.

Eligibility to receive individual financial assistance through COVID-19 pandemic relief programs was conditionally based on employment, housing composition, and income level. These relief programs specifically aimed to reduce the number of people experiencing financial difficulty during COVID-19. Thus, three additional individual-level self-reported variables were included: receipt of unemployment benefits, receipt of CTC payments, and receipt of stimulus checks (EIP).

### 2.4 Weighting and analysis

Data were weighted to the adult population living within the catchment areas using American Community Survey (ACS) 5-year estimates from the years 2015 through 2019 at the ZIP Code Tabulation Area (ZCTA) level and Public Use Microdata Area (PUMA) level to match the total number of households and the distribution of demographic characteristics. The summation of the survey weights equals to 1,056,184 residents, which is the 2019 total estimated residential adult population in BNHs (34). Unweighted and weighted frequencies and percentages were used to describe the study population. Bivariate analyses were conducted to identify covariates with significant associations with each dependent variable category. Chi-square tests were conducted and a significance level of *p* < 0.05 was used.

Two separate multinomial logistic models were run, corresponding to the research questions and independent variables of interest. First, we used a multivariable multinomial logistic regression model to examine the association between race or ethnicity and the dependent variable (i.e., “never,” “short-term,” or “prolonged” financial difficulty categories).

A second multivariable multinomial logistic regression model was developed to investigate the association between the presence of children in the household and the financial difficulty outcome. The model-adjusted risk ratio (RR) and corresponding 95% Confidence Intervals (CIs) were estimated using the predicted marginal risk ratio method, which involved the computation of the model-adjusted risk for each group in each of our main independent variables (race or ethnicity and presence of children in household) to calculate the model-adjusted risk ratio, while controlling for differences in covariate distributions between the variable categories (36).

Both models adjusted for age, gender, nativity status, educational attainment, household income level, employment status, and receipt of financial assistance programs. Key assumptions of the multinomial logistic regression model were assessed, including multicollinearity and the independence of irrelevant alternatives (IIA). Multicollinearity was evaluated using variance inflation factors (VIFs) derived from a design matrix of dummy-coded predictors. All VIFs were below the threshold of 4, indicating low risk of multicollinearity (37, 38). Model predictors were categorical and the assumption of linearity in the log-odds was not applicable. Although formal statistical tests of the IIA assumption (e.g., Hausman-McFadden) were not conducted, we determine that the outcome categories were conceptually distinct and mutually exclusive, which supports the plausibility of the IIA assumption for this model.

Missing values of bivariate model variables were excluded from the analysis, resulting in 1,321 observations for the model with race as the independent variable (2.7% missingness) and 1,340 of observations for the model with household composition as the independent variable (1.3% missingness).

#### 2.4.1 Instruments

All analyses were carried out using SAS Enterprise Guide 7.115 (SAS Institute Inc., Cary, North Carolina, USA) and SAS-Callable SUDAAN (SAS Institute Inc., Cary, North Carolina, USA).

## 3 Results

Table 1 describes the sample and bivariate results comparing the characteristics of each type of respondent: the whole sample, those who never experienced the financial difficulties of interest, those who experienced at least one financial difficulty since March 2020 but recovered, and those who were still experiencing financial difficulty in October 2021. Participants identified as either Latino or Hispanic (*N* = 508), Black non-Latino (*N*=435), White non-Latino (*N*=258), Asian non-Latino (*N*=73), or Other or Multi-racial non-Latino (*N*=47). The majority of participants identified as women (*N*=968), being born in the United States (*N*=853), reported receiving EIP during the pandemic (*N*=938), and reported that no children younger than 18 lived in their household (*N*=850).

**Table 1:** Characteristics of respondents by experience of financial difficulty, COVID-19 community recovery survey, *N* = 1,358.

	**Total *N* = 1,358**	**Never experienced any financial difficulty since March 2020**	**Experienced any financial difficulty since March 2020**	***p*- value**	**Still experiencing financial difficulty in October 2021**	***p*- value**
**Catchment area**				0.152		0.379
Harlem	523 (39%)	329 (23%)	178 (22%)		106 (19%)	
Bronx	434 (32%)	194 (35%)	220 (44%)		150 (45%)	
Brooklyn	401 (30%)	237 (42%)	146 (35%)		92 (36%)	
**Age group**				**< 0.001**		0.509
18–24 years	51 (4%)	21 (7%)	27 (9%)		17 (10%)	
25–44 years	565 (42%)	303 (45%)	242 (50%)		159 (51%)	
45–64 years	443 (33%)	229 (28%)	197 (32%)		129 (32%)	
65+ years	291 (22%)	201 (20%)	76 (10%)		42 (8%)	
**Race or ethnicity**				**< 0.001**		0.091
Latino or Hispanic	508 (38%)	225 (38%)	256 (51%)		159 (47%)	
Black, non-Latino	435 (33%)	232 (43%)	190 (41%)		131 (46%)	
White, non-Latino	258 (20%)	208 (14%)	45 (4%)		23 (3%)	
Asian, non-Latino	73 (6%)	48 (4%)	18 (3%)		13 (4%)	
Other or Multi-racial, non-Latino	47 (4%)	27 (2%)	20 (1%)		12 (1%)	
**Gender (as self-selected)**				0.641		0.579
Woman	968 (72%)	537 (52%)	391 (56%)		251 (56%)	
Man	362 (27%)	209 (47%)	140 (42%)		87 (41%)	
Transgender man, Transgender woman, Non-binary person, gender not mentioned	23 (2%)	12 (2%)	10 (2%)		8 (3%)	
**Birthplace**				0.061		**0.011**
Within U.S.	853 (63%)	515 (63%)	322 (54%)		214 (61%)	
Outside U.S.	420 (31%)	198 (31%)	189 (41%)		112 (34%)	
Within a U.S. territory	74 (6%)	40 (6%)	30 (5%)		20 (5%)	
**Individual educational attainment**				**< 0.001**		0.665
Less than high school degree	169 (12%)	60 (16%)	94 (30%)		58 (28%)	
High school graduate	268 (20%)	124 (28%)	125 (30%)		76 (31%)	
Some college, technical school, Associate degree	335 (25%)	164 (25%)	162 (25%)		119 (27.5)	
4-Year College graduate	299 (22%)	193 (17%)	97 (8%)		60 (8%)	
Graduate degree or professional degree	284 (21%)	216 (14%)	66 (6%)		35 (6%)	
**Children in household**				**< 0.001**		0.231
No	850 (63%)	551 (74%)	278 (47%)		165 (44%)	
Yes	490 (37%)	197 (26%)	260 (53%)		179 (56%)	
**Household income level**				**< 0.001**		0.241
< 200% Federal poverty level	661 (52%)	260 (46%)	359 (75%)		247 (77%)	
≥200% Federal poverty level	601 (48%)	444 (54%)	151 (25%)		84 (23%)	
**Initial employment status**				**< 0.001**		**< 0.001**
Employed	645 (48%)	409 (48%)	217 (41%)		108 (32%)	
Unemployed	175 (13%)	52 (12%)	118 (23%)		96 (28%)	
Not in labor force	417 (31%)	253 (35%)	144 (24%)		96 (25%)	
More than 1 category	118 (9%)	46 (6%)	64 (12%)		48 (15%)	
**Lost work, reduced hours, pay decrease**				**< 0.001**		**0.008**
No	603 (60%)	404 (76%)	188 (42%)		108 (35%)	
Yes	394 (40%)	140 (24%)	236 (58%)		161 (65%)	
**Received unemployment benefits**				**< 0.001**		**< 0.001**
No	1,039 (78%)	633 (85%)	379 (69%)		227 (61%)	
Yes	287 (22%)	113 (16%)	160 (31%)		118 (39%)	
**Received child tax credit**				**0.010**		**0.034**
No	1,066 (80%)	621 (85%)	408 (75%)		252 (71%)	
Yes	260 (20%)	125 (15%)	131 (25%)		93 (30%)	
**Received stimulus check**				**< 0.001**		0.209
No	388 (29%)	195 (26%)	175 (38%)		110 (35%)	
Yes	938 (71%)	551 (74%)	364 (62%)		235 (65%)	
**Death of immediate family member**				0.255		0.650
No	1,181 (93.6%)	705 (95%)	476 (92.4%)		304 (92%)	
Yes	75 (6.4%)	35 (5%)	40 (7.6%)		27 (8%)	
**Death of extended family member**				0.051		0.130
No	1,017 (78.5%)	621 (82%)	396 (74.7%)		240 (72%)	
Yes	239 (21.5%)	119 (18%)	120 (25.3%)		91 (28%)	

*p*-values derived from Wald chi-square test.

**Figure 3:**
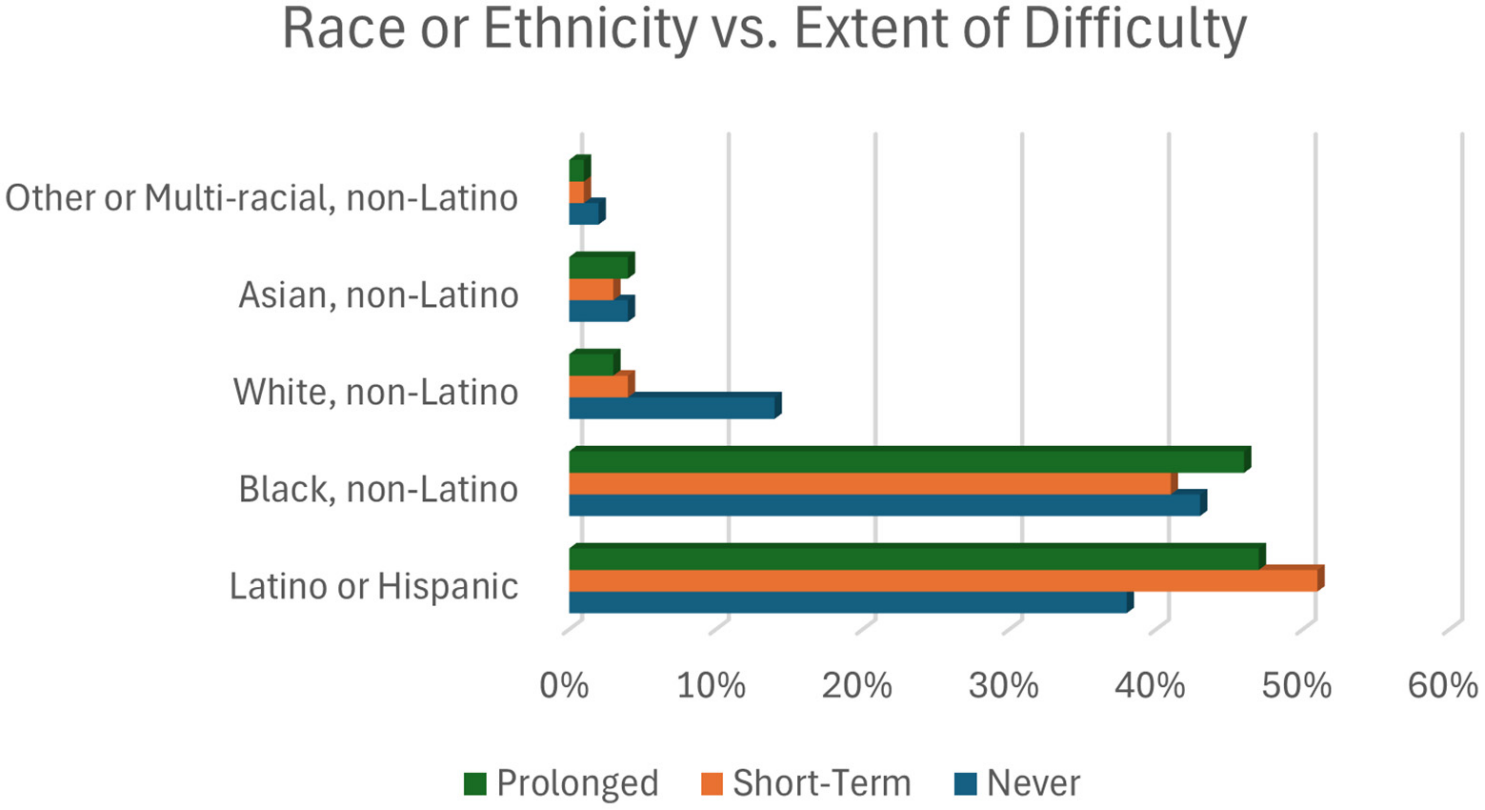
Percent of participants experiencing each of the three types of financial difficulty.

Table 1 also presents the prevalence of experience with financial difficulty due to the pandemic based on the characteristics of respondents. In the bivariate analysis, age group, race or ethnicity, educational attainment, presence of children in the household, household income level, employment status, loss of work, receipt of unemployment benefits, receipt of child tax credit, and receipt of stimulus checks were significantly associated with the experience of any financial difficulty since March 2020 (Table 1, Figure 3). Among those who reported experiencing any financial difficulty, half were aged 25–44 years, and a majority identified as either Black non-Latino or Latino or Hispanic (92%), had children in the household (53%), reported living 200% below the federal poverty level, and had lost work or wages due to the pandemic (58%). Among those who experienced any financial difficulty due to the pandemic, 41% reported being employed during panel registration in Spring 2020, while by the time of the survey done 18 months later, 58% reported a loss of work or wages due to the pandemic. Additionally, numerous respondents reported experiencing the death of at least one extended (21.5%) or immediate (6.5%) family member.

According to the bivariate analysis, prolonged financial difficulty (i.e., still experiencing financial difficulty in October 2021) was significantly associated with employment status, loss of work, receipt of unemployment benefits and receipt of child tax credits. For those who continued to experience financial difficulty, 65% lost their job or had a reduction in pay during COVID-19. Prolonged financial difficulty was significantly associated with U.S. nativity, (*p* = 0.011), where those respondents who were born in the US disproportionately reported prolonged financial difficulty.

The results of multivariable multinomial logistic regression models testing the association between race or ethnicity and type of financial difficulty experienced and household composition and type of financial difficulty experienced are presented in Tables 2, 3, respectively. The risk ratio (RR) of never experiencing a financial difficulty comparing Black respondents to White respondents was 0.75 (95% CI: 0.61, 0.92). That is, Black respondents were less likely to avoid financial hardship.

**Table 2:** Relative Risk Ratios (RR) and 95% confidence intervals (CI) for the association between race or ethnicity and COVID-related experiences with financial difficulty among participants in the COVID-19 Community Recovery study, *N* = 1,150.

**Race or ethnicity**	**Never experienced financial difficulty**, ***N*** = **666**		**Experienced short-term financial difficulty**, ***N*** = **170**		**Experienced prolonged financial difficulty**, ***N*** = **314**	
	**RR**	**(95% CI)**	**RR**	**(95% CI)**	**RR**	**(95% CI)**
White, non-Latino	1.00	—	1.00	—	1.00	—
Black, non-Latino	**0.75**	**(0.61, 0.92)**	0.76	(0.41, 1.40)	**2.19**	**(1.35, 3.53)**
Latino or Hispanic	0.80	(0.64, 1.00)	0.86	(0.48, 1.54)	**1.88**	**(1.12, 3.16)**
Asian, non-Latino	0.80	(0.58, 1.09)	0.47	(0.15, 1.50)	**2.34**	**(1.31, 4.20)**
Other or Multi-racial, non-Latino	0.73	(0.48, 1.09)	1.14	(0.47, 2.75)	1.81	(0.92, 3.57)

*p*-values derived from comparison to the group that Never experienced any of the identified financial difficulties.

Model adjusted for age; gender; nativity status; educational attainment; presence of children in household; household income level; employment status; receipt of unemployment benefits, child tax credits and stimulus checks. Bold font indicates that the point estimates is statistically significant at *p*-value < 0.05 since the confidence intervals do not include the null. The model-adjusted RR and corresponding 95% CIs were estimated using the predicted marginal risk ratio method.

**Table 3:** Relative risk ratios (RR) and 95% confidence intervals for the association between the presence of children in household and COVID-related experiences with financial difficulty among participants in the COVID-19 community recovery study, *N* = 1,150.

**Child(ren) aged under 18 in household**	**Never experienced financial difficulty**, ***N*** = **666**		**Experienced short-term financial difficulty**, ***N*** = **170**		**Experienced prolonged financial difficulty**, ***N*** = **314**	
	**RR**	**(95% CI)**	**RR**	**(95% CI)**	**RR**	**(95% CI)**
No children in the household	1.00	—	1.00	—	1.00	—
At least one child in the household	**0.68**	**(0.53, 0.86)**	1.26	(0.81, 1.96)	**1.50**	**(1.11, 2.03)**

Model adjusted for age; gender; nativity status; educational attainment; race and ethnicity; household income level; employment status; receipt of unemployment benefits, child tax credits and stimulus checks. Bold font indicates 95% confidence intervals that do not include the null value of 1.0. The model-adjusted RR and corresponding 95% CIs were estimated using the predicted marginal risk ratio method.

Conversely, the risk of experiencing prolonged financial difficulty was increased for all other racial or ethnic groups compared to White respondents: two times higher for Black and Asian respondents (RR = 2.19, 95% CI: 1.35, 3.53, RR = 2.34, 95% CI: 1.31, 4.20, respectively) and 1.88 times higher for Latino or Hispanic respondents (RR = 1.88, 95% CI: 1.12, 3.16) (Table 2).

**Figure 4:**
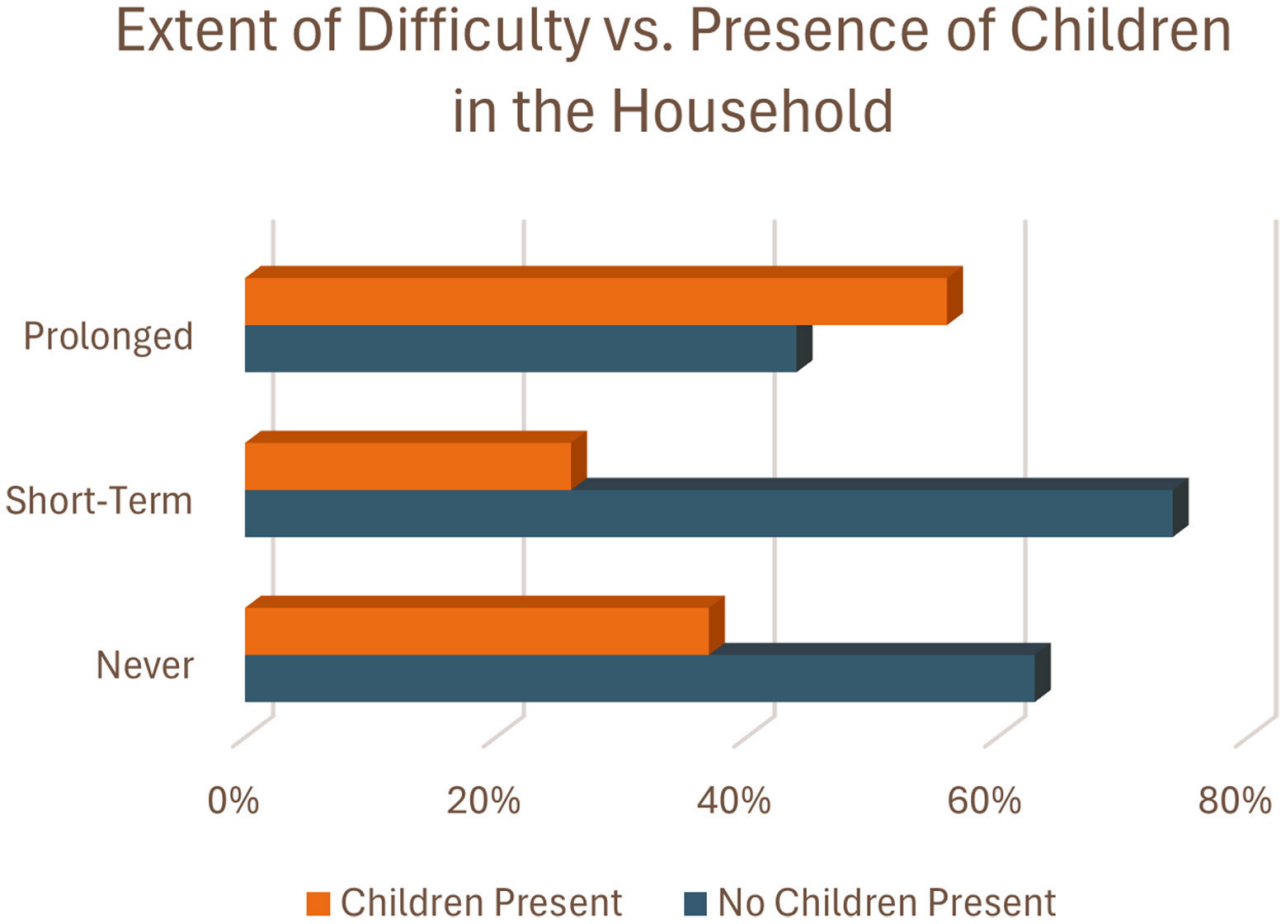
Percent of participants reporting children in their household within each difficulty category.

When looking at household composition, specifically the presence of children in the household, BNH residents with at least one child in the household were much less likely to avoid financial difficulty (RR = 0.68, 95% CI: 0.46, 0.79) compared to BNH residents with no children in the household (Table 3). Additionally, BNH residents with at least one child in the household had about 1.5 times the risk of experiencing prolonged financial difficulty (RR = 1.50, 95% CI: 1.11, 2.03) compared to BNH residents with no children in the household (Table 3, Figure 4).

## 4 Discussion

In this study of adults living in NYC's BNH areas, we found that residents identifying as Latino, Black, or Asian, people with children, and individuals who lost work disproportionately experienced financial difficulties during the COVID-19 pandemic. These groups were also less likely to recover from financial difficulties, thus experiencing them long-term. These racial and ethnic disparities persisted in the analysis even after accounting for emergency supplemental income benefits that were designed to be equitably disbursed. These characterized groups have already been demonstrated to have worse health outcomes related to COVID-19, and certain daily burdens seem to have only been worsened in BNH catchment areas by the economic impacts of COVID-19.

### 4.1 Households with children

We found that households with children were more likely than households without to experience prolonged financial difficulty despite multiple forms of financial benefits being strategically distributed to children's households.

A possible explanation for failure to recover from financial difficulties is the premature discontinuation of such funds. After the raise in CTC and other special disbursals to households with children, the U.S. Census Bureau reported a significant drop in the number of households with children reporting unmet financial needs by summer 2021 (16). However, they later reported that after the inflated CTC payouts ended in December 2021, there was a 5% rise in the number of households with children reporting difficulty meeting household expenses by February 2022—just 2 months later (39). This indicates that baseline CTC payouts may not be adequate to meeting household needs and that families may benefit from expansion of eligibility.

Adequacy of childcare has been a social determinant of health for children and their families during COVID-19. This is because, in addition to the perennial barriers to meeting childcare needs (like inadequate social support, prohibitive costs, limited facility capacity), parent work hours that misalign with conventional childcare facility business hours (40), even more barriers surfaced during the pandemic: facility closures (due to both safety precautions and untenable business expenses), a mass exodus of workers from the childcare industry, and more children (especially older children) needing care when schools closed (41, 42). In densely populated cities, pandemic conditions even impacted parents' ability to let their children go out and play (43). CTC payouts largely covered childcare and early education programming during the pandemic (41) and their continuation would have been beneficial for families with children.

The evolving childcare crisis is co-constituted with precarious employment and material hardship. Employment data from the Bureau of Labor Statistics show that the tri-state area lost 13,960 childcare workers from 2020 to 2022 (44, 45). So, even when families eventually received childcare payment vouchers, many could not actually find services to meet their needs (46). Both the existing and the emergent barriers disproportionately burden women, Latino households, Black households, and low-income families (47, 48).

In response, as of 2024, New York State has implemented the Child Care Assistance Program (CCAP) publicly funded assistance for childcare to families receiving family assistance and other low-income families funded through the Child Care and Development Block Grant (49). Eligible families may receive childcare at low or no cost through CCAP.

While the analysis did not account for the number of children present in the household, we hypothesize that hardship may be increased with more children or others needing full time care in the household. Additional research is needed to investigate the ratio of children to working adults in the household and the ages of children compared to prevalence of financial difficulties and inform future financial interventions to protect these families.

### 4.2 Complicating factors

Pandemic-related financial policies during the study period of the survey fielding fluctuated significantly and make it complicated to connect the observed hardship to any particular lack of aid. For example, one survey question asked if participants had trouble paying fares for public transportation, but standard bus fare was paused for the first 6 months of the pandemic and then was reinstated, which may have influenced the response rate to this question. It makes it difficult to do a clear comparison of time periods with and without bolstered financial supports, but what it does make clear is that if the supports were held steady in place, it may have made it more likely for people to receive the maximum benefit and help stimulate the local economy.

We believe that slow and complicated systems to disburse emergency funds contributed to the persistence of economic hardship. For example, stimulus checks were distributed based on prior-year tax returns (50). So, those who had a significant reduction in household income due to circumstances like precarious employment or even the death of household members may not have received the full benefit amount if their prior-year income was above the thresholds for receipt.

In another example, P-EBT benefits were retroactively applied to cover nutrition expenses incurred starting March 2020, but distribution was highly complicated and staggered from May 2020 through September 2020 (51, 52). This might have helped some families recover monies that were used for vital food expenses, but retroactive funds cannot undo the potential damage of 5 months of the hunger, stress, debt, and adverse health outcomes derived from economic deprivation. More research must be done on food insecurity issues that were negatively impacted or created by these financial obstacles.

In our findings, BNH residents born in the U.S. were more likely to experience financial difficulties than those born elsewhere. This community-level finding contradicts national-level studies, such as one that showed U.S. households with immigrant mothers had significantly higher rates of economic hardship than households with U.S.-born mothers during COVID (53) and one that showed increased financial stress and housing insecurity for immigrant communities in the Midwestern U.S. (54). We originally predicted that immigrant households would have higher rates of financial hardship due to a lack of explicit protections for undocumented immigrants in the CARES Act (55) and a rapid influx of immigration policies in the U.S. during the COVID era (56), but we hypothesize that social connection within immigrant communities in the BNH Catchment Areas might have been a protective factor.

### 4.3 Diminished financial safety nets

Race and household composition were already aligned with financial stress prior to COVID-19, where Black and Latino households with children had higher relative rates of financial difficulty. In COVID-19, pandemic conditions may have destabilized households with precarious financial situations even further. Now that many of the ameliorative financial safety nets have expired or been reduced, the same financial straits will likely be recreated for vulnerable, often racialized, populations.

Social vulnerability was a major predictor to U.S. areas becoming COVID-19 hotspots (57). Socially vulnerable populations, such as children and low-income families, have been found to be both more likely to die during an emergency and less likely to recover financially after an emergency (58). Following the 2002 severe acute respiratory syndrome (SARS) outbreak caused by acquisition of the related virus SARS-CoV-1, epidemiologists rightly predicted that the state of global socioeconomics would make the next pandemic even more devastating (59, 60). Disadvantageous social standing has become a global source of physical and mental health stress during COVID-19 and beyond (61–63).

There is evidence to suggest that financial situations have already worsened for many. Unemployment in BNH Community Districts rose to 10% by 2022 (64). Twenty-nine percent of respondents to the quarterly HOP that was fielded at the same time as our survey said their level of financial stress was “Overwhelming” or “Above Average” (HOP15). One year later (October to November 2022), that number rose to 41% (HOP18). The American Psychological Association's Stress in America Report shows that parents remain twice as likely to report many financial stressors and related mental health impacts (65).

Inflated unemployment benefits were provided through several mechanisms to individuals who lost work during COVID-19, but people who lost work were still unable to recover from financial difficulties. Many of these individuals can be presumed to have precarious employment in which they were underemployed prior to reported lost work. This may include already living below the poverty line while previously employed, living on tipped wages, working in poorly regulated work settings (such as domestic work, or working “off-the-books” getting paid in cash) or having unstable or too few billable working hours to meet their needs, based on census data for the areas in which participants live. Moments of crisis can result in sudden unregulated reorganization of labor (10), as occurred during COVID-19.

U.S. States with stronger safety nets had lesser impacts of stress on residents' mental health during COVID-19 (66), but social safety nets in the U.S. are being actively dismantled (24). Delays in fund distribution and premature discontinuation of funds resulted in avoidable hardship to those in need, so protocols must be written for the immediate disbursal of emergency funds to these vulnerable populations in future public health emergencies. The unequal distribution of resources and power in the U.S. are the result of government policy and those policies continue to predispose people to harm through economic deprivation (25).

### 4.4 Limitations to interpretation and generalizability

This study was powered to obtain reliable estimates in BNH neighborhoods using timely data collected from people actually living in these places and can help support continued reach to these areas even as they change over time. However, there are some limits to our interpretation of results and generalizability of results.

We assessed multicollinearity using variance inflation factors (VIFs) calculated from a dummy-coded design matrix; all VIFs were below 4, indicating no serious multicollinearity. The assumption of linearity in the log-odds was not applicable, as all included predictors were categorical. Although we did not perform a formal statistical test of the independence of irrelevant alternatives (IIA) assumption, the outcome categories in our model are conceptually distinct and mutually exclusive, which aligns with best practices for assuming IIA in applied multinomial regression (38).

Due to the mechanism of structural racism, the inclusion of socioeconomic status variables like income, educational attainment, or employment status may be on the causal pathway between race or ethnicity and relationship. That is, socioeconomic variables are usually highly correlated to race and difficult to untangle, and so are likely to yield correlated results.

The condition of having prolonged financial difficulty is limited by both our restricted characterization of financial difficulty, and its definition being relative to the time of the survey. Thus, it does not account for the length of time between the first instance of financial difficulty and the cut-off date for recovery at the time of the survey. For example, at registration, 44% of our sample group identified as being unemployed or outside the labor force and were not included in the “lost work or wages” group. However, there was no significant change in relative risk when lost work was removed from the model. Further, disaggregating financial difficulty into short-term and prolonged conditions potentially limited our ability to evaluate likelihood of recovery from financial difficulty.

In U.S. communities demographically similar to BNH catchment areas, poor reach of public health strategies was a driver of SARS-CoV-2 transmission (67). In contrast, the NYC Health Department deployed targeted public health information, outreach, and resources to BNH communities during COVID-19. Because of this and differences in welfare regimes, such as the existence of national health insurance and varying packages of emergency pandemic relief, these findings may not be generalizable to vulnerable communities in U.S. States or territories outside of New York or in international contexts.

## 5 Conclusion

BNH strategies are designed to confront spatialized environmental predispositions to poor health outcomes; and yet we see that Black, Latino, and Asian residents still have highest risk of economic deprivation among all racial groups even within these deprived areas. These results highlight how systemic racism exacerbated pre-existing economic inequality during the pandemic and indicate that relief measures were not enough to stave off long-term financial difficulties brought on by such an emergency. There may be a need for additional and prolonged economic supports needed in these areas despite the end of the emergency. Regular financial supports, rather than just incidental or emergency financial supports, may strengthen economic stability in vulnerable populations who have structural barriers to accumulating protective assets: those with precarious employment, Black and Latino residents, immigrants, and households with children.

Long-term studies have yet to be conducted on this topic due to the recentness of the pandemic, but other studies have had similar findings (68) and this analysis further illustrates just some of the inequitable financial impacts of COVID-19 despite efforts to alleviate economic challenges through state and federal emergency benefits and resources. If these emergency funds were being relied upon for fundamental needs, it was harmful to remove them from the economy. While the emergency disbursal of resources to underserved communities provides relief in which individuals may be able to maintain health and wellbeing, this is a small step in the work required to dismantle racial and socioeconomic inequities in health outcomes. People living in under-resourced communities are often praised for their resilience and tenacity, but under our current economic structures where health outcomes are correlated so closely to socioeconomics, resilience in the face of a public health emergency depends on the financial situations people live in on a daily basis. New Yorkers' material needs must first be met to approach true health equity.

## Data Availability

The datasets presented in this article are not readily available due to privacy restrictions. The data that support the findings of this study are available from authors upon reasonable request to the corresponding author and with permission of NYC Department of Health and Mental Hygiene.
